# Mortality of the Army

**Published:** 1858-07

**Authors:** 


Mortality of the Army.

Dr. Guy,* in the eloquent lecture in hand, gives some very clear and forcible expositions of his subject. We had the pleasure of hearing the lecture, and have still further pleasure now in recommending it in its printed form to our readers. One fact which we herewith quote is of great interest. It refers to the mortality of the Fire Brigade. The deaths of the London Fire Brigade corps, between the ages of forty and sixty years, number seventy per ten thousand per annum. The deaths of the Infantry of the line number by the same scale, one hundred and eighty-seven per annum; of the Foot Guards, two hundred and four; of the Household Cavalry, one hundred and ten; and of the Dragoon Guards and Dragoons, one hundred and thirty-three. Having stated these calculations, Dr. Guy proceeds thus to speak of the Fire Brigade:

"Now, this is the class of men of whom I said just now that I would give you some account. I am indebted for the particulars of their occupation and consequent mortality to Mr. Charles Frederick Browne, the surgeon to the force; and I am happy to see a gentleman here this afternoon (Mr. Braidwood, the superintendent), who can confirm the statements I am about to make.

"The ages of the firemen range from twenty to sixty and upwards; and there is one man now in the service in his seventieth year, quite able to take his turn of duty with the rest. The men are carefully selected, full three-fourths of them having been men-of-war's men. The duties they have to perform are by no means light; for each man, on the average, has been on duty at the stations, or on the watch on premises damaged by fire, three days and three nights, of twelve hours each, in every week of the past year. This is exclusive of attendance to clean the engines and tools, and keep the hose in order, and of a sort of engine drill for the younger men twice a-week. The men have also to attend and work at fires, where they are in the midst of intense heat, steam,

* The Mortality of the British Army. By W. A. Guy, M.B. London: Renshaw. 1858.

and smoke, saturated with water, and obliged to stand in elevated situations exposed to severe and cutting winds, so that the men are often seen in winter literally encrusted with ice. They are sometimes called out by fires, or alarms of fires, as many as four times in a night. But, notwithstanding this hard duty and extreme of exposure, the rate of mortality among the men is highly favourable. For the first thirteen years of the establishment, the deaths were at the rate of 96 per 10,000; while for the last twelve years they had fallen to 70 per 10,000. But these calculations include deaths by accident, which, in spite of the perilous nature of the employment, have not exceeded 44 in 10,000 in the whole period of twenty-five years. The higher mortality of the early period is attributed, and probably with justice, to less careful selection; but the moderate rate prevailing throughout the whole period of twenty-five years is evidently to be attributed to the unusual care and attention bestowed on the comforts and health of the men, who live either at the stations or in houses provided by the establishment, and subject to careful inspection. The management is in the hands of a committee, appointed by the several Fire Insurance Offices, who pay the men liberally, give them plenty of warm and comfortable clothing, instruct the medical officer and superintendent to look narrowly to the healthy state of the stations, and the other residences of the men, and act with promptitude and liberality on any suggestions which these efficient officers may please to make. This, I believe, is a fair and accurate account of this very healthy class of men.

"Here, then, we have a case of night work and exposure to weather certainly far exceeding in severity the night duty which the foot soldier has to perform; for I understand that the soldier is on guard only every fourth or fifth night. Yet this night-work, and this exposure to weather, being accompanied, in the case of the fire brigade, by the most scrupulous care of the health and comforts of the men, is compatible with the favourable rate of mortality shown in the table. May not the unfavourable death-rate of 204 in 10,000, prevailing among the Foot Guards, be partly accounted for by the substitution of carelessness for care?"

